# Optimization of Shea (*Vitellaria paradoxa*) butter quality using screw expeller extraction

**DOI:** 10.1002/fsn3.351

**Published:** 2016-03-02

**Authors:** Yonas A. Gezahegn, Shimelis A. Emire, Sisay F. Asfaw

**Affiliations:** ^1^ School of Chemical and Food EngineeringBahir Dar UniversityP.O. Box 26Bahir DarEthiopia; ^2^ School of Chemical and Bio‐EngineeringAddis Ababa UniversityP.O. Box 385Addis AbabaEthiopia; ^3^ Ethiopian Environment and Forest Research InstituteP.O. Box 24536Addis AbabaEthiopia

**Keywords:** Conditioning duration, die temperature, moisture content, response surface methodology, *Vitellaria paradoxa*

## Abstract

The quality of Shea butter is highly affected by processing factors. Hence, the aim of this work was to evaluate the effects of conditioning duration (CD), moisture content (MC), and die temperature (DT) of screw expeller on Shea butter quality. A combination of 3^3^ full factorial design and response surface methodology was used for this investigation. Response variables were refractive index, acid value, and peroxide value. The model enabled to identify the optimum operating settings (CD = 28–30 min, MC = 3–5 g/100 g, and DT = 65–70°C) for maximize refractive index and minimum acid value. For minimum peroxide value 0 min CD, 10 g/100 g MC, and 30°C were discovered. In all‐over optimization, optimal values of 30 min CD, 9.7 g/100 g MC, and 70°C DT were found. Hence, the processing factors must be at their optimal values to achieve high butter quality and consistence.

## Introduction

Shea (*Vitellaria paradoxa* C.F. Gaertn) is an indigenous fruit tree grows in the Sudano‐Sahelian belt, Africa. Which is approximately 5000 km long by 500 km wide from Senegal to Uganda and Ethiopia receiving 600–1400 mm of rainfall. There are two subspecies of the tree, one of which subsp. *paradoxa* extends from Senegal eastwards to the Central African Republic whilst the other subsp. *nilotica* occurs in Uganda, Northeast Zaire, Southern Sudan, and Ethiopia (Hall et al. 1996). In Ethiopia, the trees grow in Gambella region at an altitude 600 m a.s.l, in areas receiving an annual rainfall of about 900–1400 mm (Deribe 2005). The annual production of fruit is 15–30 kg/tree and the fruit weighs from 10 to 57 g that yields 3–4 kg of dry kernels. Butter of 45.2–59.1 g/100 g dw can be produced from this kernel. Shea butter has unique healing properties, particularly for dry skin and minor dermatological diseases superior to cocoa butter and other vegetable butters (Honfo et al. 2013). The sweet pulp of the fruit is widely consumed by locals and is a rich source of sugars, proteins, calcium, ascorbic acid, and iron (Maranz and Wiesman 2004).

The technologies that have been used for extracting Shea butter are manual (traditional boiling), semimechanized, mechanical (pressing/expeller), and solvent extraction. The traditional method is labor‐intensive, inefficient (20%), and lack consistency. The semimechanized method achieves extraction rate of 35–40%. While, fully mechanized method achieves extraction rate of 42–50% (USAID, 2004; FAO & CFC, 2004). As described by Matthäus (2012) mechanized methods has the advantages of low investment and maintenance costs, high butter quality, and easy to refine.

The physicochemical properties of Shea butter are closely related to the origin, genetic variation, pretreatments, processing factors, and methods of extraction (Maranz and Wiesman 2004; Divine et al. 2011; Honfo et al. 2013). The effect of processing factors such as kernel particle size, heating temperature, rotational speed, and nozzle size were reported as determining factors in butter quality. Where, butter from coarsely grounded groundnuts has lower free fatty acid value. As well, high kernel heating temperature and duration has negative impact in free fatty acid and peroxide value (Adeeko and Ajibola 1990). Olaniyan and Oje (2011) also observed inferior butter quality due to excessive burning from Shea kernel that has been heated above 90°C.

The refractive index of Shea butter at temperature of 40°C is around 1.46, however higher value between 1.670 and 1.690 was observed (Honfo et al. 2013; Okullo et al. 2010). The amount of free fatty acids in Shea butter found to be variable ranging from 2.13 to 17.03 mgKOH/kg. The hydrolysis of triacylglycerols to free fatty acids may occur due to enzyme action, heating, light and/or moisture (Fennema 1996). The other butter quality indicator is peroxide value, which shows the formation of peroxides/hydroperoxides that are the main products of unsaturated fatty acids oxidation. (O'Brien and Richard 2009). The effect of production method on peroxide value also resulted variation between 2.15 and 15.32 mEq/kg (Hee 2011). The lowest and highest peroxide values reported were 0.5 and 29.5 mEq/kg, respectively (Honfo et al. 2013). Major goal in Shea butter extraction is to find an appropriate operating settings to recover it while preserving its quality. To date, there is little information about influence of conditioning duration, kernel moisture content, die temperature, and their interaction effect on butter quality using screw expeller (Ixtaina et al. 2011).

Considering the unpredictability of Shea butter quality due to processing factors, the aim of this work was to evaluate the effect of conditioning duration, kernel moisture content and die temperature for maximum butter quality extracted using screw expeller. Mathematical models, which are useful for predicting and determining the optimum conditions for refractive index, acid value, and peroxide value were developed. This is a very important issue with regard to automatic regulation of plant operation in butter manufacturing. Furthermore, the overall optimization of the process, based simultaneously on butter quality, is also addressed for the first time.

## Materials and Methods

### Sample collection and pretreatments

Shea nuts, which were not damaged and spoiled, were collected from selected mother tree in Phugnido district, Gambella, Ethiopia. After, the pulp was removed by both scraping and boiling from the fruits the nuts parboiled and dried using sunlight for a week. The dried nuts cracked manually, sorted, washed, and dried using drying cabinet (Model: AS 100, 2003, Italy) at 50°C and 15% relative humidity. The resulting clean kernels were manually grinded (3–5 mm), then conditioned using autoclave (Model: KORIMAT KA 160, 2006, Germany) at 95°C and 2 MPa for 0, 15, and 30 min with saturated steam. The batches were put in drying cabinet at 50°C and 15% relative humidity to reach uniform moisture content of 3 g/100 g. Considering the initial moisture content and the mass of Shea kernel in each jar the required mass of distilled water was added to reach the target moisture content of 3, 9, and 15 g/100 g wet basis (w.b.) using Eq. (1) as performed by Zewdu and Solomon (2007).

(1) $$Q=\frac{W_{i}\left(M_{f}-M_{i}\right)}{\left(100-M_{f}\right)}$$

where Q is the mass of water to be added in kg; W_*i*_ is the initial mass of the sample in kg; M_*i*_ is the initial moisture content of the sample in g/100 g w.b. and M_*f*_ is the final moisture content in g/100 g w.b. The required amount of distilled water were added to each glass jars, then the samples were kept in refrigerator at 5°C (±1) for 5 days for the moisture to distribute uniformly throughout the sample. The jars were shaken at regular interval to facilitate internal moisture stabilization.

### Extraction of shea butter

Pretreated kernels were pressed in a single step using a Komet screw oil expeller (Model: CA 59 G, 2011, Germany). The expeller was set using 4 mm restriction die and 20 rpm screw speed. In each run the kernels were warmed in air tight jar at their perspective die temperature for 30 min just before pressing. The screw press was first run for 15 min without input material while heating *via* an electrical heating ring attached around the press barrel till it reached the desire temperature. Then the warmed kernels were expelled, while the running temperature was monitored with a digital thermometer inserted into the restriction die. Between each run the press chamber components were dismantled, washed, and dried. The extracted butter was collected and filtered using low speed centrifuge (Model: L‐530 Tabletop, 2012, China) at 5030*g* for 30 min. The filtered butter was labeled and frozen at −16°C for further analysis.

### Methods of analysis

Refractive index was measured according AOAC (2000) official method 921.08, using automatic digital Refractometer (Model: AR200 Reichert, 2011, USA) at 20°C. The butter sample was melted and filtered using qualitative filter paper (Model: Whatman grade 1, 2012, USA). Then it was poured till it covers the lens of refractometer. According to standard methods described by MOHFW (2005) about 2.5 g of melted and filtered Shea butter was placed in a 250 mL Erlenmeyer flask which was filled with 150 mL of neutralized 1:1 (v:v) ethanol and diethyl ether mixture solution. Then, the mixture was titrated with potassium hydroxide (0.1 N) ethanolic solution until the pink color appeared. Acid value and free fatty acid values were calculated using Eq. (2) and (3), respectively, where free fatty acid was expressed as oleic.

(2) $$\text{Acid value}=\frac{56.1\times T\times V}{W}$$

(3) $$\text{Free fatty acid}\;\left(\%\right)=\frac{\text{Acid value}}{1.99}$$

where T is normality of standardized potassium hydroxide solution; W is weight (g) of the test portion, and V is volume of KOH ethanolic solution used for titration (mL). Peroxide value was measured following AOAC International (2000), 965.33. About 5 g of melted filtered Shea butter sample was placed in 250 mL capacity Erlenmeyer flask. Then 30 mL of acetic acid‐chloroform solution (3:2) and 1 mL of potassium iodide saturated solution was added, then let stand for a minute in a dark. After adding 2 mL of starch solution as indicator and 30 mL of distilled water the resultant mixture showing dark purple to dark brown was titrated with standardized 0.01 N sodium thiosulfate solution till white color appears. Peroxide value (mEq/kg) was calculated using Eq. (4).

(4) $$\text{Peroxide value}=\frac{V\times T\times 1000}{W}$$

where V is volume of standardized Na_2_S_2_O_3_ (mL); T is exact normality of the sodium thiosulfate solution used; and W is weight (g) of the test portion.

### Experimental design and statistical data analysis

Processing factors that are conditioning duration, moisture content, and die temperature were varied into three levels (Table 1) to obtain second‐order and robust optimization model as described by Lazic (2004). According to Montgomery (2005) a combination of response surface methodology with full factorial design (3^3^) was developed to evaluate the contribution of each factors, their interaction and for optimization. Refractive index, acid value/FFA (Free fatty acids), and peroxide value were chosen as responses (dependent variables). Response surface methodology with miscellaneous design and five center points were used to evaluate 32 experimental runs.

**Table 1:** Experimental design of the study

No.	Processing factors	Levels		
		1	2	3
1	Conditioning duration (min)	0	15	30
2	Moisture content (g/100 g w.b.)	3	9	15
3	Die temperature (°C)	30	50	70

The adequacy of the model was determined by evaluating the lack of fit, coefficient of determination (*R* ^*2*^), and the Fisher test value (*F*‐value) obtained from the analysis of variance (ANOVA) that was generated by “Design‐Expert^®^” Version 7.0.0 (Stat‐Ease, Inc., Minneapolis, MN) software. Statistical significance of the model and model variables was determined at 5% probability level (*P* < 0.05). Each model was expressed in terms of coded factors and regardless of statistical insignificant terms. In spite of insignificance, factors that exhibit interaction was not eliminated from the models in order to support hierarchy.

## Results and Discussion

### Response surface modeling and optimization of RI

As presented in Table 3, at 30 min conditioning duration (CD), 3 g/100 g moisture content (MC), and 70°C DT the highest (1.4670) refractive index was obtained; and 0 min CD, 15 g/100 g MC, and 30°C DT yield lower (1.4655) value. The ANOVA of the quadratic regression models showed there was no significance in the lack of fit (*P* > 0.05) in each of the models (Table 2). The regression is statistically significant (*P* < 0.05) with a satisfactory determination coefficient (*R* ^*2*^ = 0.9845), where all the three factors were significant (*P* < 0.05).

**Table 2:** ANOVA for response surface reduced quadratic model

Response	Source	Sum of Squares	df	Mean Square	F Value	*P* ‐value Prob > F	
Refractive Index	Model	4.47E–06	4	1.12E–06	429.55	<0.0001	Significant
	A–CD (min)	2.57E–06	1	2.57E–06	987.5	<0.0001	
	B–MC (g/100 g)	2.45E–07	1	2.45E–07	94.18	<0.0001	
	C–DT (°C)	1.50E–06	1	1.50E–06	577.46	<0.0001	
	A ^ 2 ^	1.54E–07	1	1.54E–07	59.06	<0.0001	
	Residual	7.02E–08	27	2.60E–09			
	Lack of Fit	7.02E–08	22	3.19E–09			
	Pure Error	0	5	0			
	Cor Total	4.54E–06	31				
Acid value/FFA	Model	21.65	8	2.71	97.75	<0.0001	Significant
	A–CD (min)	14.22	1	14.22	513.58	<0.0001	
	B–MC (g/100 g)	4.49	1	4.49	162.32	<0.0001	
	C–DT (°C)	0.41	1	0.41	14.69	0.0009	
	AB	0.83	1	0.83	30.08	<0.0001	
	AC	0.084	1	0.084	3.02	0.0956	
	BC	0.2	1	0.2	7.34	0.0125	
	A ^ 2 ^	0.84	1	0.84	30.51	<0.0001	
	B ^ 2 ^	0.26	1	0.26	9.5	0.0053	
	Residual	0.64	23	0.028			
	Lack of Fit	0.48	18	0.027	0.88	0.6235	Not significant
	Pure Error	0.15	5	0.031			
	Cor Total	22.28	31				
Peroxide value	Model	138.96	8	17.37	12328.46	<0.0001	Significant
	A–CD (min)	4.77	1	4.77	3382.2	<0.0001	
	B‐MC (g/100 g)	48.66	1	48.66	34535.67	<0.0001	
	C–DT (°C)	7.43	1	7.43	5270.87	<0.0001	
	AB	0.11	1	0.11	79.37	<0.0001	
	AC	0.19	1	0.19	134.06	<0.0001	
	BC	11.51	1	11.51	8171.1	<0.0001	
	B ^ 2 ^	41.29	1	41.29	29309.65	<0.0001	
	C ^ 2 ^	40.78	1	40.78	28947.79	<0.0001	
	Residual	0.032	23	1.41E–03			
	Lack of Fit	0.03	18	1.66E–03	3.24	0.0988	Not significant
	Pure Error	2.56E–03	5	5.12E–04			
	Cor Total	138.99	31				

CD, conditioning duration; MC, moisture content.

(5) $$\begin{array}{cc} & \text{RI}=1.47+\left(3.78\times 10^{-4}\right)\text{CD}-\left(1.16\times 10^{-4}\right)\text{MC}\\ & \;+\left(2.88\times 10^{-4}\right)\text{DT}-\left(1.39\times 10^{-4}\right){\text{CD}}^{2} \end{array}$$

As it can be perceived from liner term coefficients in Eq. (5) the CD was the most significant factor. Moreover, the negative value in the quadratic term leads to maximum RI (1.4670) at 28 min. The response surfaces for maximum RI shows, the optimum values between 3–4 g/100 g MC, 28–30 min CD, and 70°C DT.

**Figure 1:**
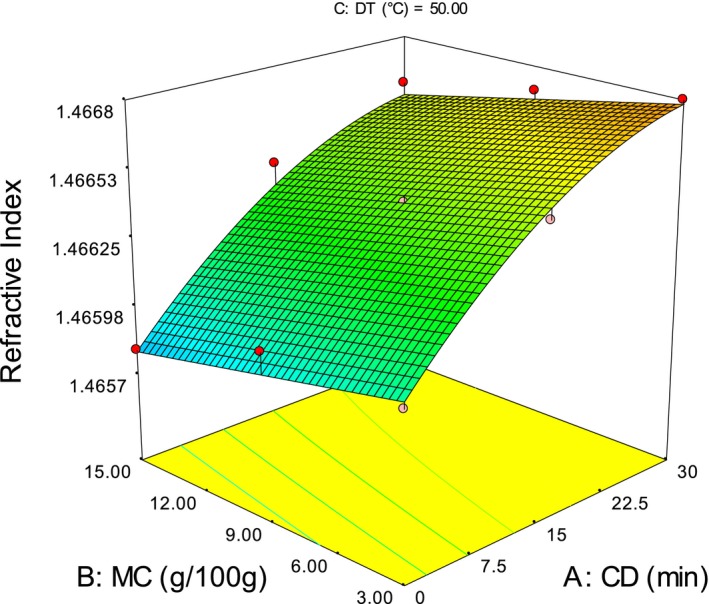
Response surface plot showing the interaction effect moisture content‐conditioning duration on refractive index.

Figure 1 depicted the longer CD related to high refractive index. This finding might be due to the inactivation of lipase enzyme at high temperature and pressure in conditioning. Since, inactivation of lipase ceases the degradation of triacylglycerols. High molecular weight and chain length might contribute for high RI. Though, after the peak value (29.94 min) the decline in RI probably resulted by the hydrolysis of triacylglycerols to free acids and other smaller molecules, by high temperature and pressure steam used for conditioning (Gunstone 2004; Damodaran et al. 2008).

Kernel moisture content shows indirect relationship with RI, this could be due to the acceleration of hydrolysis reaction that results breakdown of triacylglycerols. Besides, activity of lipase enzyme and other reactions enhanced due to high water activity which might result change in chemical structure that would reduce the refractive index (ri) (Hui et al. 2007).

The raise in die temperature results an increase in RI similar to Olaniyan and Oje (2007). This possibly caused by the inactivation of lipase enzyme at high DT, since lipase has optimal activation temperature between 35–40°C and inactivated at 70°C (Hui et al. 2007). An increase in RI as temperature rise from 30 to 50 and 70°C (Fig. 1) was expected since inactivation of lipase enzyme and simultaneous reduction in free acid formation occur. Besides, formation of complex compounds at high temperature may resulted high RI value.

### Response surface modeling and optimization of AV/FFA

As Table 3 shows the highest acid value (4.43 mg KOH/g) and FFA (2.23%) was obtained at 0 min CD, 15 g/100 g MC, and 70°C DT. While, the lowest acid value (1.24) and FFA (0.62) was obtained at 30 min CD, 9 g/100 g MC, and 30°C DT. The regression is statistically significant (*P* < 0.05) with a satisfactory determination coefficient (*R* ^*2*^ = 0.9714), where all the three factors were significant (*P* < 0.05) as shown Table 2.

**Table 3:** Some physicochemical properties of Shea butter extracted at different processing factors in screw expeller

Run	Independent variables			Physicochemical properties			
	Conditioning duration (min)	Moisture content (w.b.)	Die temperature (°C)	Refractive index	Acid value	Free fatty acids	Peroxide value
1	0	3	30	1.4658 ± 0.0005	2.46 ± 0.03	1.24 ± 0.03	4.74 ± 0.10
2	0	3	50	1.4660 ± 0.0004	2.56 ± 0.01	1.29 ± 0.01	8.53 ± 1.06
3	0	3	70	1.4663 ± 0.0007	2.73 ± 0.00	1.37 ± 0.00	7.64 ± 0.32
4	15	3	30	1.4662 ± 0.0003	1.36 ± 0.04	0.68 ± 0.04	4.94 ± 1.07
5	15	3	50	1.4665 ± 0.0005	1.27 ± 0.01	0.64 ± 0.01	9.00 ± 0.26
6	15	3	70	1.4669 ± 0.0007	1.34 ± 0.04	0.67 ± 0.04	8.24 ± 0.36
7	30	3	30	1.4665 ± 0.0008	1.33 ± 0.00	0.67 ± 0.00	5.28 ± 1.00
8	30	3	50	1.4668 ± 0.0004	1.30 ± 0.01	0.66 ± 0.01	9.37 ± 1.01
9	30	3	70	1.4670 ± 0.0006	1.28 ± 0.00	0.64 ± 0.00	8.75 ± 0.00
10	0	9	30	1.4656 ± 0.0008	2.92 ± 0.00	1.47 ± 0.00	1.56 ± 0.70
11	0	9	50	1.4660 ± 0.0007	2.87 ± 0.00	1.44 ± 0.00	4.47 ± 0.88
12	0	9	70	1.4662 ± 0.0006	3.19 ± 0.00	1.60 ± 0.00	2.64 ± 0.70
13	15	9	30	1.4662 ± 0.0008	1.68 ± 0.01	0.84 ± 0.01	1.97 ± 0.16
14	15	9	50	1.4664 ± 0.0008	1.88 ± 0.04	0.95 ± 0.04	4.95 ± 0.00
15	15	9	50	1.4664 ± 0.0008	1.89 ± 0.04	0.95 ± 0.04	4.90 ± 0.00
16	15	9	50	1.4664 ± 0.0002	1.48 ± 0.01	0.74 ± 0.01	4.95 ± 0.00
17	15	9	50	1.4664 ± 0.0002	1.89 ± 0.02	0.95 ± 0.02	4.92 ± 0.00
18	15	9	50	1.4664 ± 0.0002	1.81 ± 0.04	0.91 ± 0.04	4.90 ± 0.00
19	15	9	50	1.4664 ± 0.0002	1.97 ± 0.04	0.99 ± 0.04	4.92 ± 0.00
20	15	9	70	1.4668 ± 0.0008	2.11 ± 0.03	1.06 ± 0.03	3.26 ± 0.98
21	30	9	30	1.4664 ± 0.0004	1.24 ± 0.01	0.62 ± 0.00	2.35 ± 0.00
22	30	9	50	1.4667 ± 0.0006	1.31 ± 0.01	0.66 ± 0.00	5.48 ± 0.29
23	30	9	70	1.4670 ± 0.0004	1.28 ± 0.01	0.64 ± 0.01	3.94 ± 1.03
24	0	15	30	1.4655 ± 0.0001	3.71 ± 0.02	1.86 ± 0.02	3.15 ± 1.03
25	0	15	50	1.4658 ± 0.0004	3.69 ± 0.00	1.85 ± 0.00	5.11 ± 0.77
26	0	15	70	1.4660 ± 0.0008	4.43 ± 0.03	2.23 ± 0.03	2.22 ± 1.04
27	15	15	30	1.4660 ± 0.0006	2.24 ± 0.02	1.13 ± 0.02	3.62 ± 0.09
28	15	15	50	1.4664 ± 0.0006	2.68 ± 0.04	1.35 ± 0.04	5.67 ± 1.02
29	15	15	70	1.4666 ± 0.0008	3.03 ± 0.02	1.52 ± 0.02	2.96 ± 1.06
30	30	15	30	1.4662 ± 0.0006	1.50 ± 0.00	0.76 ± 0.00	4.20 ± 0.00
31	30	15	50	1.4666 ± 0.0008	1.56 ± 0.00	0.78 ± 0.00	6.25 ± 1.05
32	30	15	70	1.4668 ± 0.0008	1.76 ± 0.01	0.88 ± 0.01	3.70 ± 0.42

Values are means ± SD (*n* = 3).Refractive index (at 20°C); Acid value (mg KOH/g); Free fatty acid (as oleic acid); and Peroxide value (mEq/kg).

(6) $$\begin{array}{cc} & \text{AV}=1.82-0.89\text{CD}+0.50\text{MC}+0.15\text{DT}-0.26\text{CD}\;\times \;\text{MD}\\ & \;-0.08\text{CD}\;\times \;\text{DT}+0.13\text{MC}\;\times \;\text{DT}+0.34{\text{CD}}^{2}\\ & \;+0.19{\text{MC}}^{2} \end{array}$$

**Figure 2:**
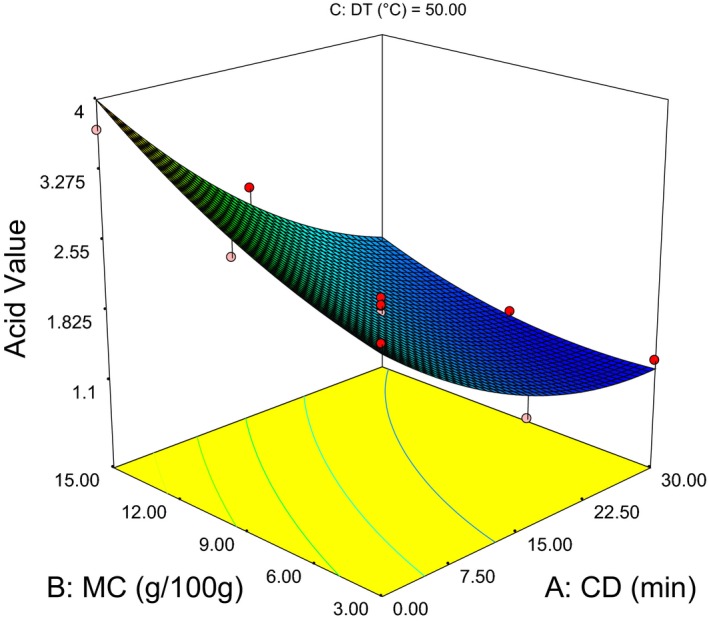
Response surface plot showing the interaction effect moisture content‐conditioning duration on acid value.

As it can be observed from liner term coefficients in Eq. (6) the CD had high impact and indirect relation with acid value. Where, the DT was the least influential factor in manipulating the acid value/FFA. The positive value in quadratic term of both CD and MC leads to a minimum value of acid value (AV) around CD = 30 min and MC = 5.5 g/100 g as shown in Figure 2. Hence, the response surface for minimum AV (1.18) shows the optimum values around 29 min CD, 5 g/100 g MC, and 65°C DT.

Since conditioning of the kernels would inactivate enzymes and microorganisms that would cause formation of free acids the reduction in AV/FFA at longer conditioning duration was expected (Bockisch 1998; Gunstone 2002). However, prolonged conditioning duration beyond the optimal value might result rise of AV. This might be caused by the hydrolysis of triacylglycerols by high temperature and pressure steam used for conditioning.

A significant effect of MC on the AV/FFA was also observed (Fig. 2). This might be due to accelerated hydrolysis reaction that results breakdown of triacylglycerols into free acids at high moisture. Also, increase in water activity favors other type of reactions that form acidic compounds like phosphates and amino acids (Nielsen 2010; Hui et al. 2007).

The direct relationship between DT and AV/FFA was similar finding to Olaniyan and Oje (2007). This probably caused by the hydrolysis of triacylglycerols at high temperature. When the temperature is beyond the optimal value (35–40°C) of lipase enzyme activity, a slight increment of AV was observed instead of decline. This fact indicates the formation of other acidic compounds like, phosphate and amino acid which did not solely depend on lipase enzyme (Hui et al. 2007; Bockisch 1998; Nielsen 2010).

### Response surface modeling and optimization of PV

The ANOVA of the quadratic regression models showed there was no significance in the lack of fit (*P* > 0.05) (Table 2). The regression is statistically significant (*P* < 0.05) with a satisfactory determination coefficient (*R* ^*2*^ = 0.9998), where all the three factors were significant (*P* < 0.05). This means that the models could be used to predict the response.

(7) $$\begin{array}{cc} & \text{PV}=4.95+0.51\text{CD}-1.64\text{MC}+0.64\text{DT}+0.09\text{CD}\\ & \;\times \text{MC}+0.13\text{CD}\times \text{DT}-0.98\text{MC}\times \text{DT}\\ & \;+2.36{\text{MC}}^{2}-2.34{\text{PT}}^{2} \end{array}$$

**Figure 3:**
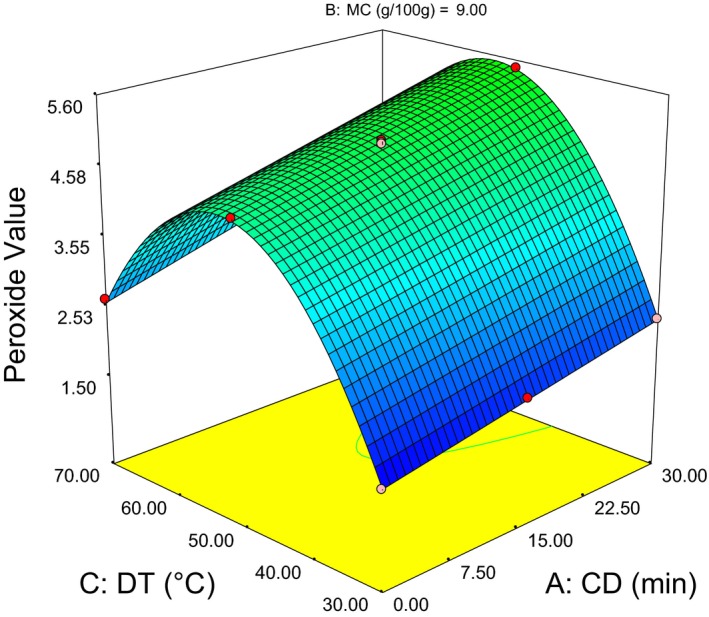
Response surface plot showing the interaction effect die temperature‐conditioning duration on peroxide value.

**Figure 4:**
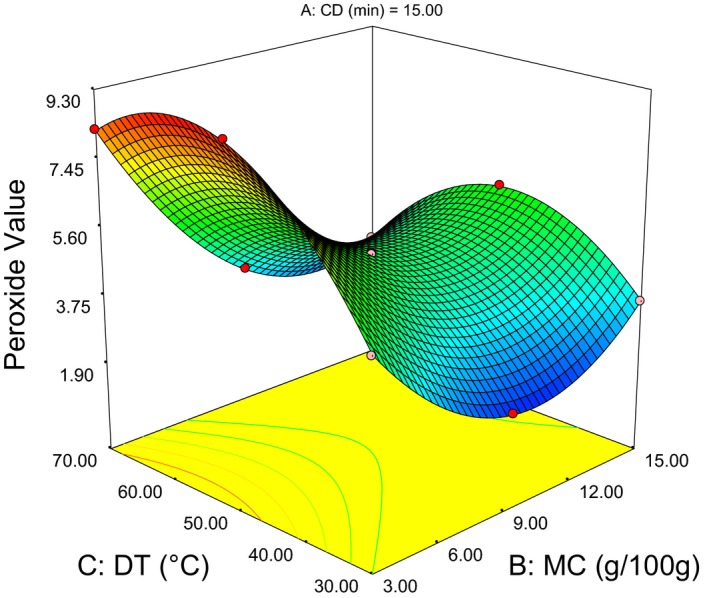
Response surface plot showing the interaction effect die temperature moisture content on peroxide value.

As presented in (Table 3), the highest (9.37 mEq/kg) peroxide value was obtained at 30 min CD, 3 g/100 g MC, and 50°C DT. While, the lowest (1.56) value was obtained at 0 min CD, 9 g/100 g MC, and 30°C DT. As it can be observed from the liner term coefficients in Eq. (7), MC has negative value and almost three times greater in magnitude than other factors. This implies it is the most significant factor with inverse relation. Besides, the positive coefficient in MC quadratic term leads to a minimum (1.54) peroxide value (PV) at 10 g/100 g MC (Fig.  4). As well the negative coefficient in DT quadratic term leads to a maximum (9.5) PV at 55°C (Fig. 3). Hence, the response surfaces for minimum PV (1.54) show the optimum around 0 min CD, 10 g/100 g MC, and 30°C DT.

Figure 3 illustrate rise of PV as CD increases, this possibly caused by the reaction between oxygen from steam used for conditioning and unsaturated fatty acids within the kernel that formed peroxides and hydroperoxides (O'Brien and Richard 2009; Gunstone 2002). Figure 4 indicates decline of PV as the MC increases up to 10 g/100 g and a rise in PV was observed beyond the optimum value. Similar phenomena also discussed by Fennema (1996) that suggested addition of water to a very dry sample is believed to bind hydroperoxides, interfering with their decomposition and thereby hindering the progress of oxidation. In addition, this water dilutes metal ions that catalyze oxidation, apparently reducing their effectiveness. On the other hand, addition of water beyond the optimum will increase oxygen solubility and by allowing macromolecules to swell it exposes more catalytic sites that will increases rates of oxidation.

Moreover, less moisture in the kernel results precipitation of minerals in the butter than being solubilized and expelled out with press cake. This might accelerate the oxidation reaction at lower kernel moisture content. However, high moisture in the kernel beyond the optimal (10 g/100 g) might favors oxidative reaction between oxygen from water and unsaturated fatty acids within the kernel (Damodaran et al. 2008; Bockisch 1998; Gunstone 2002).

The increase in die temperature up to 55°C resulted rise of peroxide value, and after that decline was observed. This fact also explained by Afaf (2003) that the decomposition of hydroperoxides and other side reactions are minimized around 60°C, so that all type of peroxides can be quantified. This might gave peak value of the graph, where highest peroxide value was measured and there was no decomposition of hydroperoxides. The decline in peroxide value beyond 55°C was in agreement with the explanation given by Matthäus (2012) that explains at high temperature the hydroperoxides were completely decomposed and the peroxide value decreases rapidly.

### Overall optimal conditions

**Figure 5:**
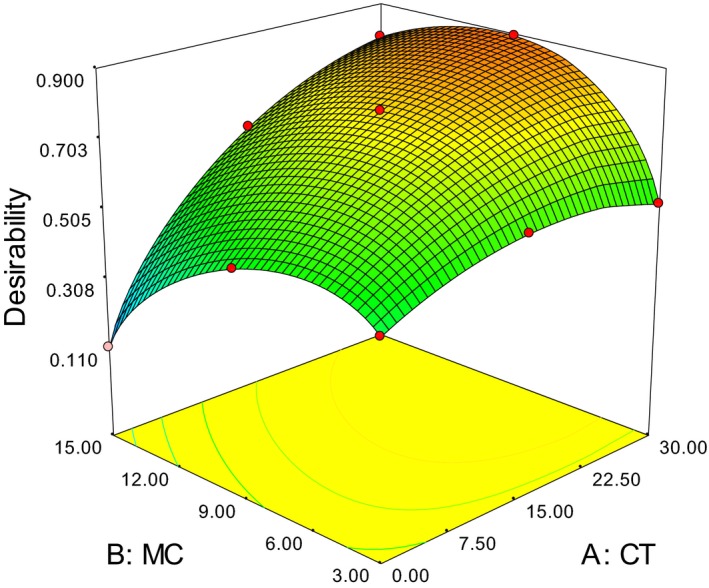
Response surface representation of overall optimization.

As discussed in previous sections optimal conditions for each response were discovered separately. Yet, it can be observed that the optimal values of each factors do not match for all responses. Hence, to find an overall optimal condition, a response surface optimization covering all the three responses (refractive index, acid value, and peroxide value) was applied. Optimization criteria were as follows: maximize refractive index; minimize acid value and peroxide value, where all responses given equal weight. Optimal CD of 30 min, MC between 9.66–9.73 g/100 g and DT at 70°C were found (Fig. 5).

## Conclusion

In conclusion, the interaction effects of conditioning duration, kernel moisture content, and die temperature were investigated using a combination of response surface methodology with full factorial design. In the study different model equation was used to express each response; and all three factors were significant in all the cases. The optimum values for maximum refractive index (30 min CD, 3 g/100 g MC, 70°C DT), minimum acid value/FFA (30 min CD, 5 g/100 g MC, 65°C DT), and minimum peroxide value (0 min CD, 10 g/100 g MC, 30°C DT) were obtained. In overall all optimization, giving equal weight for all factors, 30 min CD, 9.7 g/100 g MC, and 70°C DT were obtained. Hence, the optimum operating conditions must be kept to achieve consistence and high butter quality.

## Conflict of Interest

None declared.
